# Supplementary material for: Study protocol for a randomized controlled clinical trial of a multifaceted cognitive training program using video games in childhood cancer survivors
Source: PLoS One. 2025 Sep 2;20(9):e0314118. doi: 10.1371/journal.pone.0314118 (PMC12404409; doi:10.1371/journal.pone.0314118)
Supplement: Attachment — Submitted filename: Response to reviewers. June 26, 2025.docx [file pone.0314118.s002.docx]

Dear Academic Editor and Reviewers,

We sincerely thank you for your thorough evaluation of our manuscript entitled "Randomized controlled clinical trial with a multifaceted cognitive training program using video games in childhood cancer survivors." We appreciate the insightful and constructive comments provided by the reviewers and editorial team, which have helped us substantially improve the quality and clarity of the manuscript.

Please find below our point-by-point responses to all concerns raised. For each comment, we have provided the original reviewer/editor request in bold, followed by our response.

**Academic editor’s requirements:**

**1. Please ensure that your manuscript meets PLOS ONE's style requirements, including those for file naming.**

The manuscript has been modified to fully comply with PLOS ONE’s formatting and style requirements.

**2. Your ethics statement should only appear in the Methods section.**

The ethics statement has been moved to the Methods section and removed from all other parts of the manuscript.

**3.** [**Please include captions for your Supporting Information files at the end of your manuscript.**](http://track.editorialmanager.com/CL0/http:%2F%2Fjournals.plos.org%2Fplosone%2Fs%2Fsupporting-information./1/010f01966d309a0d-59127e40-23a8-43e5-a2d8-29a0e879d894-000000/nWnN1XGsl7yFq5jqFv76KSvCKtwilA49cOrcEkKXllM=208)

Captions for the Supporting Information files are now provided at the end of the manuscript and have been labeled according to the journal’s Supporting Information guidelines. All Supporting Information files have been renamed to correspond with these labels.

**4. Remove copyrighted logos from the uploaded protocol.**

All copyrighted institutional logos were removed.

**Reviewer #1 – Major comments**

**1. Sample Size Justification and Power Calculation.**

The reviewer is correct in noting that G*Power does not support true mixed-effects models. The initial sample size estimation was performed using a fixed-effects model with repeated measures ANCOVA (2 groups × 3 time points), assuming:

- Effect size: f = 0.25 (moderate)
- α = 0.05
- Power (1−β) = 0.95
- Software: G*Power 3.1.9.4

However, we acknowledge that our primary planned analysis involves a mixed-effects model with repeated measures, which allows for modeling intra-subject correlations. The use of a fixed-effects model for power estimation could lead to a slight underestimation of the required sample size, particularly if the intra-class correlation (ICC) is high.

To address this, we have reviewed the potential impact of including intra-subject correlation in the sample size estimation. According to published guidelines and simulations for mixed-models with similar designs, assuming ICC values between 0.3 and 0.6 (common for neurocognitive outcomes), the impact on required sample size remains moderate, particularly given our model with three repeated measures.

For transparency, we have now added a discussion in the revised manuscript noting this limitation and reporting the sample size estimations under different ICC scenarios. For example, assuming ICC = 0.5, and similar parameters, simulations suggest that a total sample size of 42–48 subjects would achieve equivalent power, which we consider feasible, given our planned recruitment.

**2. Statistical Analyses and Model Assumptions.**

We have now clarified and specified the statistical models in the revised manuscript. In summary:

Mixed ANCOVA (main analysis):

- Between-subjects factor: Group (Intervention vs. Control)
- Within-subjects factor: Time (T0, T+3, T+6)
- Covariates: Age, sex, baseline cognitive scores, cancer diagnosis type, treatment modality (e.g., CNS radiation, chemotherapy with neurotoxic agents)
- Interaction terms: Group × Time interaction is explicitly modeled to assess differential changes across time by group.

Multiple linear regressions (exploratory):

- Predictors: treatment group, age, baseline cognitive profile, psychosocial indicators
- Outcomes: neurocognitive test scores, neuroimaging markers, inflammatory biomarker changes

All models will be checked for assumptions (normality, homoscedasticity, multicollinearity) and complemented with appropriate effect size indices (e.g., omega squared, R², Cohen’s f).

We have added explanations of variable inclusion in the updated Methods section.

**3. Missing Data and Attrition Handling.**

Thank you for pointing this out. As a longitudinal study, we anticipated some degree of attrition and missing data.

We have now clarified the following:

- Missing data will be handled using Multiple Imputation (MI) under the assumption of missing at random (MAR), which is implemented using chained equations.
- Sensitivity analyses will be conducted by comparing results with and without imputation and using complete-case analyses.
- If patterns suggest non-random missingness, we will perform sensitivity models including inverse probability weighting or pattern-mixture models as appropriate.

We have added a new paragraph in the Statistical Analysis subsection that explicitly describes this.

**4. Randomization and Blinding Issues.**

The study is partially blinded. Randomization is stratified by age and implemented using REDCap. Clinical follow-up staff will not be blinded, but neuropsychological and neuroimaging assessors will remain blinded to the group allocation to minimize outcome bias.

We have clarified these aspects in the revised “Randomization and Blinding” subsection and discussed the potential for bias and mitigation strategies for unblinded roles.

**5. Interpretation of Biomarker Results**

We appreciate the reviewer’s thoughtful observation regarding the interpretation of cytokine data. It is indeed well recognized that inflammatory cytokines such as IL-6 and TNF-α exhibit substantial inter- and intra-individual biological variability, influenced by circadian rhythms, age, infection status, physical activity, and psychological stress.

In our study, cytokine levels will be measured at standardized time points and using highly sensitive and validated multiplex immunoassays, in order to minimize pre-analytical variability. Furthermore, the samples will be processed in a centralized laboratory to ensure consistency in handling and assay performance.

Although no universally accepted clinical thresholds exist for defining abnormal cytokine levels in pediatric cancer survivors, we will rely on previously published pediatric reference ranges (de Jager et al., 2009; de Lima et al., 2019) and internal control group distributions to aid interpretation. As such, we did not pre-specify rigid clinical cut-offs, but rather aim to assess within-subject changes over time and between-group differences in the direction and magnitude of the inflammatory response.

Importantly, we plan to complement group-level comparisons with Reliable Change Index (RCI) analyses and regression-based approaches to identify whether individual biomarker changes correlate with neurocognitive outcomes, helping to contextualize biological relevance even in the presence of high variability.

We have added a paragraph in the Discussion section to clarify our strategy for interpreting the biomarker results and the limitations inherent to cytokine variability.

**Reviewer #1 – Minor comments**

**Clarify the Hypothesis Testing Strategy**

Each primary outcome (neuropsychological test scores, neuroimaging variables, and biomarker levels) will be analyzed using separate statistical models, given the different data structures and measurement characteristics of each domain. We will not apply a global multivariate test across all outcome types. Within each domain, appropriate multivariate procedures will be considered where applicable (e.g., MANOVA for groups of related cognitive outcomes), but primary hypothesis testing will be conducted through parallel domain-specific analyses.

**Address Potential Bias in Patient Selection.**

We agree that the inclusion criterion of patients 1–8 years post-treatment may introduce survivor bias. This aspect is now explicitly acknowledged as a limitation in the Discussion section. We have added a note clarifying that our findings may not generalize to all pediatric cancer survivors, particularly those with early relapse or severe neurological sequelae that prevent participation. However, we believe that the selected time window reflects a clinically meaningful survivor population in which cognitive late effects are often the most prominent and intervention is still viable.

**Ethical and Transparency Considerations.**

The ethical protocol approved by the institutional review board ensures anonymization and secure storage of all patient data. We plan to share de-identified raw datasets, including neuroimaging and biomarker data, upon reasonable request, after the publication of the main findings. This will be noted in the revised “Ethics and dissemination” section. Data sharing will comply with GDPR and institutional policies.

**Reviewer #2**

**1. The introduction lacks a strong justification**

To strengthen the rationale for the study, we have added a new paragraph at the end of the Introduction section. This paragraph highlights the current gaps in the literature regarding the variability and unclear incidence of neurocognitive sequelae in pediatric cancer survivors, lack of correlation with neuroimaging findings, and absence of systematic screening protocols or engaging interventions specifically designed for this population. The added paragraph clarifies the unmet clinical and research needs that the present study aims to address.

**2. Additional details are required on the selection criteria**

We expanded the selection criteria section to include the inclusion and exclusion criteria for the healthy control group. This clarifies the basis for the comparison of neuropsychological and neuroimaging assessments and improves the transparency of our recruitment strategy.

**3. The statistical analysis methods need more explanation.**

The Statistical Methods section has been thoroughly revised to address this and the comments of other reviewers.

**4. Figures and tables should be more clearly labeled and referenced appropriately in the text.**

We have re-labeled all figures and tables according to the journal’s guidelines and are appropriately referenced in the text.

**5. Some results are presented without adequate discussion on their implications.**

We have reviewed the manuscript and expanded the discussion in selected areas to better contextualize the preliminary findings and their potential implications, particularly in relation to neurocognitive outcomes and inflammatory markers.

**6. Ensure consistency in data reporting and units of measurement.**

We appreciate the reviewer’s comment. We have carefully revised the manuscript to ensure that all results are reported consistently, with standardized units of measurement and terminology across tables, figures, and text.

**7. Clarify any contradictory findings and provide a more detailed discussion in relation to previous studies.**

We have revised the Discussion section to address the apparent inconsistencies.

**8.There are sections with ambiguous phrasing and grammatical inconsistencies**

The manuscript has been thoroughly proofread and corrected for its clarity and grammar.

**9. The conclusion section should better summarize key findings and their impact.**

We have revised the conclusion to summarize the study objectives more clearly and highlight the potential clinical impact of the proposed intervention, as follows:

*“Increased survival in pediatric cancer patients highlights the urgent need to diagnose and treat the neuropsychological effects of both the disease and its treatments. Although this condition has been widely studied, we do not have standardized screening protocols or evidence-based cognitive rehabilitation tools currently available in routine clinical practice. By evaluating neuropsychological performance, neuroimaging variables, and blood biomarkers, this study will help to determine the prevalence, severity, and potential mechanisms of neurocognitive impairment in childhood cancer survivors. In parallel, it will assess the feasibility and effectiveness of a multifaceted cognitive training platform using video games, which, if beneficial, could offer a novel, engaging, and scalable strategy for improving long-term outcomes in this population”.*

**10. Recommendations for future research should be expanded.**

Thank you for this suggestion. We have added a brief statement at the end of the Discussion, outlining potential future research directions based on the current study design and objectives, as follows:

*“Future studies should explore the long-term sustainability of cognitive improvements following this type of intervention, and examine whether earlier implementation, closer to the end of treatment, may enhance its effectiveness. In addition, further research is needed to validate the relationship between changes in cognitive function and neuroimaging or biomarker profiles in larger multicenter cohorts.”*

**11. Ensure that all references follow the required journal format.**

All references have been revised to comply with the journal guidelines.

**12. Some figures could be improved for clarity and readability.**

Figure 1 has been enlarged to enhance clarity.

**13. Define all abbreviations when first mentioned.**

All abbreviations are now defined upon first mention in the text.

**Reviewer #3**

**1. why do you exam these specific immune and and inflammatory biomarkers?**

Thank you for this question. The selected cytokines (IL-2, IL-4, IL-6, IL-10, IL-17a, TNF-α, IFN-γ, and IL-1 receptor antagonist), along with lymphocyte subpopulations, were chosen based on prior evidence linking immune dysregulation and systemic inflammation to cancer-related cognitive impairment (CRCI). In particular, IL-6 and TNF-α have been repeatedly implicated in neuroinflammation and cognitive dysfunction in both cancer and non-cancer populations (Cheung et al., 2015; Janelsins et al., 2014).

IL-10 and IL-1RA are anti-inflammatory markers that may reflect compensatory or regulatory immune responses. The inclusion of Th1, Th2, and Th17 cytokines provides a broader immunological profile that may help elucidate the inflammatory balance in pediatric cancer survivors.

This panel also aligns with the cytokines evaluated in previous studies of cognitive outcomes in pediatric and adult oncology, supporting comparability and hypothesis-driven interpretation.

We have added a paragraph in the Methods section explaining this.

**2. who was included to the control group?**

The selection criteria section has been completed to explain the inclusion of patients in the control group.

**3. who was responsible for neuropsychological evaluation?**

Neuropsychological evaluations will be performed by clinical neuropsychologists from the Department of Psychiatry, Clinical Psychology, and Mental Health of the hospital. This information has been included in the “Variable and instruments” subsection of the manuscript.

**4. what is adherence evaluation?**

Table 1 shows the different times of the study in which adherence to treatment will be verified. This will be performed using the systems included in some of the gaming platforms, and by telephone/video calls to the participants. This information is detailed now in table.

**5. the number of patients should be bigger**

The sample Size Justification and Power Calculation have been revised and completed in the Statistical Methods section regarding all reviewers’ comments.

**6. The CNS survivors should be analysed seperately.**

We agree with the reviewer that patients with CNS involvement may present with distinct neurocognitive profiles and should be carefully considered. In our analysis plan, we have specified that the diagnostic group (CNS vs. non-CNS tumors) will be included as a covariate in all primary models. Additionally, exploratory subgroup analyses will be performed to compare the cognitive and neuroimaging outcomes between CNS and non-CNS survivors, where the sample size allows. We have clarified this in the revised Statistical Methods section.

We are grateful for the reviewers’ thoughtful feedback and believe that the revised manuscript has been substantially improved. We hope that the revised version meets the journal’s expectations, and thank you for your time and consideration.

Sincerely,

Antonio Perez-Martinez
